# Speech-language disorder severity, academic success, and socioemotional functioning among multilingual and English children in the United States: The National Survey of Children’s Health

**DOI:** 10.3389/fpsyg.2023.1096145

**Published:** 2023-02-20

**Authors:** Matthew E. Foster, Ai Leen Choo, Sara A. Smith

**Affiliations:** ^1^Child and Family Studies, College of Behavioral and Community Sciences, University of South Florida, Tampa, FL, United States; ^2^Communication Sciences and Disorders, College of Education and Human Development, Georgia State University, Atlanta, GA, United States; ^3^Technology in Education and Second Language Acquisition, College of Education, University of South Florida, Tampa, FL, United States

**Keywords:** speech language disorders, severity, multilingual children, monolingual children, academic success, socioemotional functioning, bullying

## Abstract

Research points to negative associations between educational success, socioemotional functioning, and the severity of symptoms in some speech-language disorders (SLDs). Nonetheless, the majority of studies examining SLDs in children have focused on monolinguals. More research is needed to determine whether the scant findings among multilinguals are robust. The present study used parent report data from the U.S. National Survey of Children’s Health (2018 to 2020) to gain a better understanding of the impacts of SLD severity on indicators of academic success and socioemotional functioning among multilingual (*n* = 255) and English monolingual (*n* = 5,952) children with SLDs. Tests of between-group differences indicated that multilingual children evidenced more severe SLDs, had lower school engagement, and had lower reports of flourishing than English monolingual children with SLDs. Further, a greater proportion of multilingual children with SLDs missed more school days than English monolinguals. However, multilinguals were less likely to bully others or have been bullied than monolinguals. While the previous between-group differences were statistically significant, they were small (*vs* ≤ 0.08). Increased SLD severity predicted an increased number of repeated school grades, increased absenteeism, and decreased school engagement, when age and socioeconomic status were controlled. Increased SLD severity also predicted greater difficulty making and keeping friends and decreased flourishing. The effect of SLD severity on being bullied was statistically significant for the monolinguals but not multilinguals. There was a statistically significant interaction for SLD severity and sex for school engagement and difficulty making and keeping friends for monolinguals but not multilinguals. The interactions indicated that school engagement decreased more for females than for males while difficulties making and keeping friends increased more for males than females as one’s SLD severity increased. While some findings were specific to monolinguals, tests of measurement invariance indicated that the same general pattern of relations among the variables were evident across the groups of multilinguals and monolinguals. These final findings can inform the interpretation of the results from both the current and future studies, while the overall findings can inform the development of intervention programs, thereby improving the long-term academic and socioemotional outcomes of children with SLDs.

## 1. Introduction

Speech-language disorders (SLDs) interfere with one’s process of normal communication and are one of the most commonly reported disorders in childhood (Law et al., 2015). Symptoms of SLDs range from difficulties with articulation, voice, and stuttering to challenges with spoken and written language production. The reported prevalence of SLDs differs across types, varying between 1–25%, with higher rates in younger children and males (Dodd, 1995; Keating et al., 2001; McLeod et al., 2013; American Speech-Language-Hearing Association [ASHA], 2020; Irwin et al., 2022). Extant research suggests that children with SLDs face an elevated risk for academic and socioemotional difficulties relative to their peers without SLDs (e.g., National Research Council, 2009; National Assessment of Educational Progress, 2022). For example, children with speech sound disorders show lower academic skills compared to their peers without speech sound disorders, and severity is negatively correlated with reading and spelling abilities (Bird et al., 1995; Webster et al., 1997; Lewis et al., 2000). Similarly, children with developmental language delay show lower literacy (reading and writing) skills compared to children without developmental language delay (Ziegenfusz et al., 2022). The emphasis on verbal communication skills in the U.S. educational system spotlights the deficits among children with SLDs. That is, verbal communication is the primary medium of classroom instruction in the U.S, so children who have labored speech production, make articulation errors, or evidence other characteristics of SLDs, may struggle academically. Routine educational activities such as reading aloud and participating in classroom discussion may become more challenging as the severity of a child’s SLD increases; thereby leading to negative impacts on a child’s academic success (O’Brian et al., 2011).

In general, academic achievement has been found to predict college attendance and earnings in adulthood (Duckworth et al., 2012). Lower academic performance in high school is correlated with lower likelihood of college attendance and graduation, and lower wage earnings as adults (French et al., 2015). These associations may be aggravated in children with SLDs who are more likely to have academic difficulties. Children with SLDs are less likely to graduate from high school or pursue a college degree compared to children without SLDs, and symptom severity is negatively correlated with college attendance (Snowling et al., 2001; Fleming and Fairweather, 2012; Rees and Sabia, 2014). The impact of a SLD extends beyond childhood. Adults with a history of childhood SLDs are less likely to participate in the workforce but when they do, they are more likely to be unskilled manual workers, experience higher rates of discrimination and termination compared those without a history of SLD (Cronin et al., 2020; Langbecker et al., 2020). Simply put, SLDs may produce a cascading, cumulative disadvantage that begins in childhood. Consequently, determining factors that moderate academic achievement in childhood is a crucial step in supporting the needs of children with SLDs.

Children with SLDs are at higher risk for socioemotional difficulties compared to children without SLDs (e.g., Baker and Cantwell, 1982, 1987; Prizant et al., 1990; Beitchman et al., 1996). For example, children with speech language impairment are more likely to present difficulties related to socializing and internalizing compared to their typically developing peers (Redmond and Rice, 1998). Children with voice disorders express feelings of embarrassment, frustration, and anger as a consequence of difficulties using their voice (Connor et al., 2008). School-age children who stutter are up to six times more likely to have social anxiety disorder and seven times more likely to have generalized anxiety disorder compared to children who do not stutter (Iverach et al., 2016). SLDs may make it difficult to lead peers in play, participate in pretend play, resolve conflicts, engage in problem solving discussions, and provide explanations (Langevin et al., 2009). As a result, a child’s difficulty making and keeping friends may increase as the severity of their SLD worsens (Davis et al., 2002). SLDs, particularly those affecting speech, may make children more susceptible to teasing and bullying (Dockrell and Howell, 2015). Indeed, as many as 83% of children that stutter confirm being teased or bullied at school (Hugh-Jones and Smith, 1999). School-age students with SLDs (e.g., stuttering, voice disorder) report heightened apprehension toward speaking in groups, reading in class, and interpersonal conversations (Blood et al., 2001; Connor et al., 2008), which in turn, corresponded to negative attitudes to school and poorer academic performance (Blood et al., 2001).

When considering the relations between SLD severity, academic success, and socioemotional functioning, it could be important to consider a child’s biological sex and a family’s socioeconomic status (SES). The findings from several studies indicate that genetics and being male are risk factors for SLDs (e.g., Dodd, 1995; Keating et al., 2001; McKinnon et al., 2007; Harrison and McLeod, 2010; Eadie et al., 2015; American Speech-Language-Hearing Association [ASHA], 2020; Choo et al., 2022). Putative sex-related differences in cognition may protect females against SLDs. In particular, females generally outperform males in executive functioning tasks (Gur et al., 2012), and executive functioning is strongly linked to speech and academic performance (Felsenfeld et al., 2010). Therefore, sex related differences in executive function could reduce susceptibility to SLDs in females (Choo et al., 2022); however, sex was not related to SLDs in at least one other study (Fox et al., 2002). Regarding SES, some studies suggest that SLDs are more common among children from low SES backgrounds (Roberts et al., 1976; Eadie et al., 2015; National Academies of Sciences, Engineering, and Medicine, 2016). Other studies, however, have failed to show a relation between SES and the prevalence of SLDs (Beitchman et al., 1996; Harel et al., 1996; Felsenfeld and Plomin, 1997; Keating et al., 2001). Nonetheless, given the well-known relation between SES and academic success, SES is an important variable to consider in national cohort studies conducted in the U.S.

Most studies examining SLDs have focused on monolinguals (e.g., for an overview see Choo and Smith, 2020; and Kohnert and Medina, 2009). However, findings related to typically developing multilingual children and monolingual children with SLDs suggests that children with SLDs who are also multilingual may be even more vulnerable to academic and socioemotional difficulties. At least in the U.S., multilingual children, particularly those who are English-language-learners^1^ and from lower socioeconomic backgrounds, show lower academic achievement compared to monolingual English-speaking peers (Han, 2012; Callahan, 2013; however, see Padilla and Gonzalez, 2001). Children who are multilingual, who are more likely to be from racial/ethnic minority families, also experience higher levels of discrimination, acculturation stress, and bullying that negatively impact socioemotional functioning (Koo et al., 2012; Kang et al., 2014; Zeiders et al., 2016). The presence of speech-language impairments may magnify academic difficulties faced by multilingual children, which are exacerbated as their symptoms intensify. Although the extent to which SLD severity impacts the academic success of multilingual children in the U.S. is unknown, the studies discussed above suggest that greater symptom severity is linked with lower academic success (e.g., Bird et al., 1995; Webster et al., 1997; Lewis et al., 2000) and socioemotional functioning (e.g., Davis et al., 2002).

Traditionally, studies examining the skills and functioning of children with SLDs have used standardized assessments to ascertain skills and functioning (e.g., Test of Language Development; Expressive Vocabulary Test; Beitchman et al., 1996; Lewis et al., 2000; Conti-Ramsden et al., 2009). However, there is a growing number of studies that suggest parent reports are a reliable and rich source of information on academic skills, socioemotional functioning, and development in children, including those with SLDs (e.g., Hall and Segarra, 2007; Guiberson et al., 2011; McLeod et al., 2013; however, see Hauerwas and Stone, 2000). Notably, parent-identified speech difficulties at age 2 years have been found to predict speech sound disorders at age 4 years (Eadie et al., 2015). Parent-reported communication skills in preschool children with language impairment also predicted reading (based on the Woodcock Johnson Reading Mastery), writing (Test of Written Language) and math (Key Math) abilities at the 3-year follow-up (Hall and Segarra, 2007). Further, information on receptive and expressive communication collected from parents of preschool children with language impairment using the Vineland Adaptive Behavior Scales (Communication Domain) significantly correlated with performance on the Peabody Picture Vocabulary Test, Token Test for Children, and Mean Length Utterance (Hall and Segarra, 2007). A study by McLeod et al. (2013) also suggests that parent reports may offer a broader account of socioemotional functioning in children with SLDs (specifically, speech sound disorder) that correlates with disparate situational variables. In short, the challenges and strengths identified by parental reports offer a panoptic view of development and functioning in children with SLDs that is not restricted to the classroom.

To our knowledge, only one prior study has investigated multilingualism and SLDs in a large national cohort study. Choo et al. (2022) demonstrated that the prevalence of SLDs among multilingual children was lower than among English monolingual children and was higher among males than females. Choo et al. (2022) also demonstrated that being multilingual was associated with an increased likelihood of being identified with moderate or severe symptoms compared to English monolingual children). The present study extends the small body of literature focused on the intersection of SLDs and multilingualism, and directly extends Choo et al. (2022) who investigated the prevalence, severity, and risk factors for speech disorders in multilingual and English monolingual children in the U.S. using the National Survey of Children’s Health (NSCH). The present study extends Choo et al. (2022) by using the NSCH data to investigate the relations between SLD severity, academic success, and socioemotional functioning among multilingual and English monolingual children, considers the potential protective effects of sex, and controls for SES in the analyses. The results of the present study are important as they could inform treatment and the development of intervention programs.

In summary, individuals with SLDs are more likely to have lower academic success and lower socioemotional functioning, including being teased and bullied, than their non-SLDs peers. The strength of these associations likely increase as the severity of the child’s SLD increases. However, no study has examined the utility of SLD severity in predicting the academic success and socioemotional functioning of multilingual and English monolingual children. The severity of a child’s SLD may impact academic success and socioemotional functioning, which may differ for multilinguals and English monolinguals. Therefore, it could be beneficial to investigate SLD severity and its utility in predicting academic success and socioemotional functioning in multilinguals and English monolinguals while also considering the moderating effects of sex–the purpose of the present study, which was guided by the following research questions.

***Research Question #1:** Is sex, SES, and SLD severity predictive of academic success and social–emotional functioning among school age children with SLD?*

***Research Question #2:** Are the relations between sex, SES, SLD severity, academic success, and socioemotional functioning equivalent across multilingual and English monolingual school age children with SLD?*

***Research Question #3:** Does sex moderate the relations between SLD severity, academic success, and socioemotional functioning for school age children with SLD?*

## 2. Methods

### 2.1. Participants

This study used the publicly available NSCH data from the years of 2018 to 2020 (Child and Adolescent Health Measurement Initiative [CAHMI], 2022a), a nationally representative data set of children under 18 years old in the U.S.^2^ The survey has been administered annually since 2016 and monitors U.S. trends in a range of children’s health topics and well-being. Randomly selected households across the United States were mailed instructions to access an online survey; some addresses also received a paper version. For each household, data were collected from a randomly selected sample of adults and children. Information about the child was collected from an adult, typically the parent or guardian. For the purposes of the present study, participants were between the ages of 6 and 17 years who had a SLD and had data present for the primary language used in the household (*n =* 6,207). Participants 6-years of age and older were chosen because SLD typically become apparent or peak in children between 3-and 5-years of age with some children recovering from SLDs such as stuttering around 6-years of age (e.g., Ambrose et al., 1997). In addition, some survey items on the NSCH survey were not asked of parents with children younger than 6-years of age (e.g., questions related to bullying) while other survey items (e.g., flourishing) differed for children under the age of 6 years compared to children ≥6 years. Finally, some survey items changed substantially (i.e., either the question or the answer choices) since 2016 (e.g., school engagement items); thereby limiting the present study to data from 2018 to 2020. For the purposes of the present study, children of parents who reported a language other than English as the primary language spoken in the home were classified as multilingual, resulting in 255 multilingual and 5,952 English monolingual children.

### 2.2. Measures

In the following, we describe each measure used in the present study. For additional information on how the measures were conceptualized, constructed, and interpreted, please see Child and Adolescent Health Measurement Initiative [CAHMI] (2022b).

#### 2.2.1. Speech-language disorder severity

Parent-rated severity of a child’s current speech or other language disorder was measured on a 3-point scale: (1) “Does not currently have condition,” (2) “Current condition, rated mild,” (3) “Current condition, rated moderate/severe.”

#### 2.2.2. Academic success

Academic success was measured by three variables. One, parents were asked if their child repeated any grades since starting in kindergarten, which was a dichotomous item (i.e., “yes” or “no” response). Two, school engagement was a composite from two items on the parent survey, reflecting the frequency with which their child, cares about doing well in school and completes all their required homework. Both questions were measured on a 4-point scale: (1) “Always,” (2) “Usually,” (3) “Sometimes,” (4) “Never.” The two items were combined to create an indicator of school engagement measured on a 3-point scale: (1) “Always to both items,” (2) “Always or usually to one item or usually to both items,” (3) “Sometimes or never to both or any item.” Finally, parents were asked “During the past 12 months, about how many days did this child miss school because of illness or injury?” Missed school days were measured on a 5-point scale: (1) “No missed school days,” (2) “1–3 days,” (3) “4–6 days,” (4) “7–10 days,” (5) “11 or more days.”

#### 2.2.3. Socioemotional functioning

Socioemotional functioning was measured through four variables. One, parents were asked, “Compared to other children his or her age, how much difficulty does this child have making or keeping friends,” which was measured on a 3-point scale: (1) “No difficulty,” (2) “A little difficulty,” (3) “A lot of difficulty.” The second variable, flourishing, was based on three questions that aimed to capture curiosity and discovery about learning, resilience, and self-regulation. The survey questions asked, “How often does this child: (1) show interest and curiosity in learning new things, (2) work to finish tasks they start, and (3) stay calm and in control when faced with a challenge?” The 4-point scale for these three items was (1) “Always,” (2) “Usually,” (3) “Sometimes,” (4) “Never.” Of the 4-point scale, the “Always” or “Usually” responses rather than the “Sometimes” and “Never” responses indicated the child meets the flourishing item criteria. Thus, the responses to the three questions were recoded to create a single variable in the NSCH dataset for flourishing, measured on a 3-point scale: (1) “Meets 0–1 flourishing items,” (2) “Meets 2 flourishing items,” (3) “Meets all 3 flourishing items.” Parents were also asked about two facets of bullying. Specifically, “During the past 12 months, how often was this child bullied, picked on, or excluded by other children?” and “During the past 12 months, how often did this child bully others, pick on them, or exclude them?” The response for both questions was measured on a 5-point scale: (1) “Never (in the past 12 months),” (2) “1–2 times (in the past 12 months),” (3) “1–2 times per month,” (4) “1–2 times per week,” or (5) “Almost every day.”

#### 2.2.4. Socioeconomic status

To measure SES, the child’s household poverty level was derived from parent responses about family income. Specifically, parents were asked, “What is the income level of the household that the child lives in? Responses were coded according to federal poverty levels (FPLs), “Household income 0–99% FPL,” “Household income 100–199% FPL,” “Household income 200–399% FPL,” or “Household income 400% FPL or greater/.”

#### 2.2.5. Biological sex

To measure the child’s sex, parents were asked, “What is the child’s sex?” The only response categories were “Male” and “Female.”

### 2.3. Data analytic overview

To better understand the groups of children, preliminary analyses were conducted, including tests of between group differences on key demographic characteristics and the frequency distributions for the observed variables. With the exception of child age, which used a *t-*test and Hedges *g*, the chi-square test of independence (*χ*^2^) was used to determine if there is statistically significant relationship between the each observed variable and group, while *Cramer’s v* provided an effect size estimate of the relationship. In contrast to *g*, which is unbounded, *v* ranges between 0 and 1, and was interpreted according to the following conventions: <0.10 (negligible association), 0.10 to 0.19 (weak association), 0.20 to 0.39 (moderate association), 0.40 to 0.59 (relatively strong), ≥0.60 (strong) (Rea and Parker, 1992). Hedges *g* was interpreted according to Cohen’s (1988) conventions for small (0.20), medium (0.50), and large (0.80). Finally, histograms, descriptive statistics, *q-q* plots, and correlations of the observed variables were examined to ensure that the variables met the assumption of normality to use regression within a path analytic framework.

To gain a better understanding of the associations between the SLD severity, academic success, and socioemotional functioning, we used multigroup path analyses that accounted for the effects of the child’s sex and family SES using Mplus (version 8.6). Because households were randomly selected to participate in the NCHS from across the U.S., a nested structure to account for non-independence of the child outcomes was not needed. After examining main effects, path analyses that examined the interaction of SLD severity by sex were estimated. Model 1 included the three academic outcomes. Model 3 included the four socioemotional outcomes. Models 2 and 4 were extensions of the Models 1 and 3 that included the interaction of SLD severity and sex. Estimates of *R*^2^, the variance in the outcome accounted for by the set of predictors in Models 1–4, were interpreted according to Cohen’s (1988) conventions for small (0.01–0.08), medium (0.09–0.24), and large (≥0.25). To determine if the relations among the variables were equivalent between the group of multilingual and English monolinguals, tests of measurement invariance were used. Specifically, Models 1–4 were estimated again while constraining the parameter estimates (i.e., regression estimates, *β*, correlations among outcomes, intercepts, and residual variances) from each Model to equality across the two groups. The tenability of the fully constrained Models was judged relative to values for commonly used fit indices (i.e., the *χ*^2^ test of model fit, RMSEA, CFI, SRMR) as discussed in Marsh et al. (2004). Although lack of significance (*p* > 0.05) for the *χ*^2^ test of model fit indicates acceptable model fit (Kline, 2011), if additional fit criteria were adequate, a significant *χ*^2^ test of model fit was considered acceptable because the statistical significance of this test can be influenced by the sample size (Kline, 2011). To avoid biased estimates and loss of statistical power from listwise deletion of cases missing data, all multigroup path analyses were estimated using full information maximum likelihood estimation with robust standard errors (MLR) in Mplus (Version 8.6). Estimates of *R*^2^, the variance in the outcome were interpreted according to Cohen’s (1988) conventions for small (0.01–0.08), medium (0.09–0.24), and large (≥0.25).

## 3. Results

### 3.1. Preliminary analyses

#### 3.1.1. Demographic characteristics

The sample’s demographic characteristics are displayed in Table 1. As reported elsewhere (e.g., Dodd, 1995; McLeod et al., 2013), there were more males than females in the sample, *χ*^2^(1) = 716.59, *p* < 0.001, *v = *0.34^3^. However, the proportion of male and female children were comparable for multilinguals and English monolinguals *χ*^2^(1) = 1.56, *p =* 0.21, *v = *0.02. In contrast to English monolinguals, multilingual children where from homes where English was not the primary language spoken (*χ*^2^(2) = 6,207.00, *p* < 0.001, *v =* 1.00). A greater proportion of multilingual children were born outside of the U.S., *χ*^2^(1) = 159.44, *p* < 0.001, *v = *0.16, and were Hispanic or Asian, *χ*^2^(6) = 959.26, *p* < 0.001, *v = *0.39, compared to monolinguals. The groups also differed in age, *t*(277.17) = 5.41, *p* < 0.001, *g = *0.34, and SES, *χ*^2^(3) = 100.08, *p* < 0.001, *v = *0.13. On average, multilingual children were 1.19 years younger than monolinguals and from lower SES backgrounds. Although similar proportions of multilingual and English monolinguals had special education plans (*χ*^2^(1) = 2.80, *p* > 0.05, *v = *0.02), the groups differed with regard to some comorbid conditions. Relative to monolinguals, the proportion of multilingual children with Down syndrome (*χ*^2^(1) = 18.74, *p <* 0.001, *v = *0.06), intellectual disability (*χ*^2^(1) = 15.88, *p <* 0.001, *v = *0.05), learning disability (*χ*^2^(1) = 5.97, *p <* 0.05, *v = *0.03), and autism (*χ*^2^(1) = 8.09, *p <* 0.05, *v = *0.04) was greater than expected by chance; however, the magnitudes of the differences were small.

**Table 1:** Sample’s demographic characteristics by group with tests of between group differences.

	Multilinguals (*n* = 255)	English Monolinguals (*n* = 5,952)	*x*^2^(*df*)	Effect Size
Age in years (*m*, sd)	10.22 (3.43)	11.40 (3.50)	5.41 (277.17)^***1^	Hedges *g* = 0.34
Federal poverty level (FPL)			100.08 (3)^***^	Cramer’s *v* = 0.13
Household income 0–99% of the FPL	68 (27%)	820 (14%)		
Household income 100–199% of the FPL	84 (33%)	1,070 (18%)		
Household income 200–399% of the FPL	72 (28%)	1874 (31%)		
Household income 400% of the FPL or greater	31 (12%)	2,188 (37%)		
Sex			1.56 (1)	Cramer’s *v* = 0.02
Female (*n*, %)	75 (29%)	1,974 (33%)		
Male (*n*, %)	180 (71%)	3,978 (67%)		
Race/Ethnicity			959.26 (6)^***^	Cramer’s *v* = 0.39
White (*n*, %)	26 (10%)	4,280 (72%)		
Black (*n*, %)	8 (3%)	422 (7%)		
Asian (*n*, %)	42 (17%)	172 (3%)		
Hispanic (*n*, %)	174 (68%)	595 (10%)		
Hawaiian/Pacific Islander (*n*, %)	2 (<1%)	8 (<1%)		
American Indian (*n*, %)	0 (0%)	54 (1%)		
Multiracial (*n*, %)	411 (7%)	3 (1%)		
Home Language			6,207.00 (2)^***^	Cramer’s *v* = 1.00
English (*n*, %)	0 (0%)	5,952 (100%)		
Spanish (*n*, %)	165 (65%)	0 (0%)		
Other	90 (35%)	0 (0%)		
Born in the U.S. (*n*, %)	207 (81%)	5,733 (96%)	159.44 (1)^***^	Cramer’s *v* = 0.16
Special education plan (*n*, %)	174 (68%)	4,340 (73%)	2.80 (1)	Cramer’s *v* = 0.02
Comorbid conditions				
Cerebral palsy	9 (3%)	149 (3%)	1.06 (1)	Cramer’s *v* = 0.01
Down syndrome	14 (6%)	104 (2%)	18.74 (1)^***^	Cramer’s *v* = 0.06
Tourette syndrome	2 (1%)	46 (1%)	0.001 (1)	Cramer’s *v* = 0.00
Behavioral or conduct problems	81 (32%)	1,742 (30%)	0.74 (1)	Cramer’s *v* = 0.01
Developmental delay	137 (54%)	2,816 (48%)	3.93 (1)	Cramer’s *v* = 0.03
Intellectual disability	49 (29%)	667 (11%)	15.88 (1)^***^	Cramer’s *v* = 0.05
Learning disability	124 (49%)	2,441 (41%)	5.97 (1)^*^	Cramer’s *v* = 0.03
Autism	70 (28%)	1,202 (20%)	8.09 (1)^*^	Cramer’s *v* = 0.04
ADHD	60 (24%)	1,730 (29%)	3.48 (1)	Cramer’s *v* = 0.02

Column percentages reported; Data for race/ethnicity missing for 31 participants and for sex 21 participants. Percents do not equal 100% due to rounding. ^1^T-test (df) conducted equal variances not assumed. ^*^p < 0.05, ^***^p < 0.001.

#### 3.1.2. Frequency distributions of the observed variables

There were significant associations between home language status and six of the eight observed variables and responses (see Table 2). Compared to the English monolinguals, parents of multilingual children reported their child’s SLD symptoms as more severe, *χ*^2^(2) = 35.82, *p* < 0.001, *v = *0.08, their school engagement as lower, *χ*^2^(2) = 9.21, *p* < 0.01, *v = *0.04, and they were less likely to be described as flourishing, *χ*^2^(2) = 20.57, *p* < 0.001, *v = *0.06. The pattern for missed school days also differed between multilinguals and English monolinguals, *χ*^2^(4) = 12.96, *p* < 0.01, *v = *0.05, which may be due to the proportion of multilinguals that did not miss school. Finally, multilinguals were less likely to bully others, *χ*^2^(4) = 18.82, *p* < 0.001, *v = *0.06, or have been bullied by others than English monolinguals, *χ*^2^(4) = 28.49, *p* < 0.001, *v = *0.07. While statistically significant, it is important to remember that the previous associations were interpreted as negligible according to the conventions posited by Rea and Parker (1992).

**Table 2:** Frequency distributions for the observed variables from the NSCH 2018–2020, *n* = 6,207.

Observed variables and responses	Frequency (Valid %)		*x*^2^(*df*)	Cramer’s *v*
	Multilinguals (*n* = 255)	English monolinguals (*n* = 5,952)		
Severity of child’s speech or language disorder			35.82 (2)^***^	0.08
Does not currently have condition	73 (29%)	2,538 (43%)		
Current condition, rated mild	83 (33%)	1,992 (34%)		
Current condition, rated moderate/severe	95 (73%)	1,320 (23%)		
Academic Success				
Repeated any grade			0.42 (1)	0.01
Yes	36 (15%)	769 (13%)		
No	213 (86%)	5,128 (87%)		
School engagement			9.21 (2)^**^	0.04
Always to both items	64 (26%)	1,660 (28%)		
Always or usually to one item or usually to both items	79 (32%)	2,240 (38%)		
Sometimes or never to both or any item	108 (43%)	1,997 (34%)		
Missed school days			12.96 (4)^**^	0.05
No missed school days	75 (30%)	1,314 (23%)		
1–3 days	81 (33%)	2,406 (41%)		
4–6 days	43 (17%)	1,083 (19%)		
7–10 days	22 (9%)	566 (10%)		
11 or more days	27 (11%)	478 (8%)		
Socioemotional Functioning				
Difficulty making and keeping friends			1.15 (2)	0.01
No difficulty	110 (44%)	2,802 (48%)		
A little difficulty	81 (33%)	1,884 (32%)		
A lot of difficulty	57 (23%)	1,219 (21%)		
Flourishing			20.57 (2)^***^	0.06
Meets 0–1 flourishing items	123 (48%)	2,047 (35%)		
Meets 2 flourishing items	50 (20%)	1,334 (23%)		
Meets all 3 flourishing items	82 (32%)	2,540 (43%)		
Bullied, picked on, excluded others in the past 12 months			18.82 (4)^***^	0.06
Never (in the past 12 months)	215 (86%)	4,354 (74%)		
1–2 times (in the past 12 months)	28 (11%)	1,111 (19%)		
1–2 times per month	5 (2%)	218 (4%)		
1–2 times per week	1 (<1%)	130 (2%)		
Almost every day	1 (<1%)	62 (1%)		
Been bullied, picked on, excluded by others in past 12 months			28.49 (4)^***^	0.07
Never (in the past 12 months)	131 (52%)	2,197 (37%)		
1–2 times (in the past 12 months)	79 (32%)	2,065 (35%)		
1–2 times per month	24 (10%)	796 (14%)		
1–2 times per week	7 (3%)	491 (8%)		
Almost every day	9 (4%)	321 (6%)		

Column percentages reported. Percentages may not equal 100% due to rounding. ^**^p < 0.01; ^***^p < 0.001.

#### 3.1.3. Descriptive statistics for the sample

Descriptive analyses and visual inspections of the ordinal data revealed a floor effect for parent report for ‘bullied others’ within the group of multilinguals (i.e., *kurtosis* = 17.79; see Table 3). However, the skewness and kurtosis values for ‘bullied others’ among the English monolinguals and the remaining measures for both groups of children did not exceed the recommended threshold of |3.00| and |10.0| for skewness and kurtosis, respectively (Weston and Gore, 2006). Given that MLR estimation in Mplus is well known for its ability to reliably estimate models with non-normal data, we did not transform the ‘bullied others’ variable for either group of children.

**Table 3:** Sample’s descriptive statistics by group.

	Multilinguals (*n* = 255)					English monolinguals (*n* = 5,952)				
	Missing	*M*	*SD*	*Skew*	*Kurtosis*	Missing	*M*	*SD*	*Skew*	*Kurtosis*
Socioeconomic status	0 (0%)	2.26	0.99	0.23	−1.00	(0%)	2.91	1.04	−0.55	−0.91
Severity of child’s speech or language disorder	4 (2%)	2.09	0.82	−0.16	−1.48	102 (2%)	1.79	0.79	0.38	−1.28
Repeated any grade	6 (2%)	1.14	0.35	2.03	2.15	55 (1%)	1.13	0.34	2.20	2.82
School engagement	4 (2%)	1.82	0.81	0.33	−1.40	55 (1%)	1.94	0.79	0.10	−1.37
Missed school days	7 (3%)	2.38	1.30	0.74	−0.51	105 (2%)	2.40	1.17	0.77	−0.21
Difficulty making and keeping friends	7 (3%)	1.79	0.79	0.40	−1.30	47 (1%)	1.73	0.78	0.51	−1.19
Flourishing	0 (0%)	1.84	0.88	0.32	−1.65	31 (0%)	2.08	0.88	−0.16	−1.68
Bullied, picked on, excluded others in the past 12 months	5 (2%)	1.18	0.51	3.73	17.79	77 (1%)	1.37	0.75	2.52	6.92
Been bullied, picked on, excluded by others in past 12 months	5 (2%)	1.74	1.00	1.61	2.44	82 (1%)	2.09	1.15	1.01	0.20

#### 3.1.4. Correlations

All bivariate relations were linear and in the expected direction (see Table 4). The pattern of correlations was relatively similar for the group of multilinguals and English monolinguals. Although some of the correlations were not statistically significant for the multilinguals, they could be practically meaningful as some of the correlations (e.g., between the child’s sex and flourishing) were stronger for the multilinguals compared to English monolinguals but only flagged as statistically significant for the English monolinguals. Indeed, a test of measurement invariance that held the unstructured covariance matrices to equality fit the data well, suggesting the magnitude of the correlations across the groups are equivalent (see Table 5, Model 1).

**Table 4:** Correlations among all variables multilinguals (bottom panel) and English monolinguals (top panel).

	1.	2.	3.	4.	5.	6.	7.	8.	9.	10.
Child’s assigned sex at birth	–	0.04^**^	−0.02	−0.02	0.15^***^	0.03^*^	−0.06^***^	0.06^***^	−0.03^*^	0.02
Socioeconomic status	0.04	–	−0.16^***^	−0.10^***^	0.15^***^	−0.08^***^	−0.06^***^	0.18^***^	−0.06^***^	−0.09^***^
Severity of child’s speech or language disorder	0.01	−0.01	–	0.17^***^	−0.27^***^	0.11^***^	0.35^***^	−0.33^***^	0.04^**^	0.14^***^
Repeated any grade	0.01	0.01	0.16^**^	–	−0.13^***^	0.05^***^	0.12^***^	−0.14^***^	0.01	0.07^***^
School engagement	0.12	0.02	−0.32^***^	−0.11	–	−0.16^***^	−0.37^***^	0.56^***^	−0.21^***^	−0.23^***^
Missed school days	0.06	−0.09	0.26^**^	0.10	−0.18^**^	–	0.17^***^	−0.16^***^	0.07^***^	0.19^***^
Difficulty making and keeping friends	−0.06	0.10	0.40^**^	0.11	−0.46^**^	0.21^***^	–	−0.47^***^	0.20^***^	0.44^***^
Flourishing	0.08	0.12	−0.32^**^	−0.13^*^	0.63^**^	−0.14^*^	−0.46^**^	–	−0.20^***^	−0.27^***^
Bullied, picked on, excluded others in the past 12 months	−0.01	−0.01	0.01	0.03	−0.08	−0.01	0.01	0.03	–	−0.40^***^
Been bullied, picked on, excluded by others in past 12 months	−0.05	−0.08	0.09	0.04	−0.15^*^	0.03	0.22^***^	−0.11	0.23^***^	–

^*^p < 0.05, ^**^p < 0.01, ^***^p < 0.001.

**Table 5:** Tests of measurement invariance for multilinguals and English monolinguals children.

Model	*x*^2^(*df*)	BIC	SSABIC	RMSEA	CFI	SRMR
Unstructured covariance matrices	9.27 (9)	72,412	72,314	0.003	1.00	0.010
Academic functioning main effects model	20.61 (18)	36,232	36,175	0.007	0.998	0.022
Academic functioning main effects with interaction model	23.51 (21)	36,252	36,185	0.006	0.998	0.021
Social–emotional functioning main effects model	138.50 (26)^***^	57,249	57,166	0.038	0.978	0.054
Social–emotional functioning main effects with interaction model	95.63 (28)^***^	57,241	57,140	0.028	0.987	0.054
Unstructured covariance matrices – SES and SLD severity freely estimated	4.71 (8)	72,201	72,315	0.000	1.00	0.006

^***^p < 0.001.

#### 3.1.5. Path analyses

##### 3.1.5.1. Academic success

The results of the multigroup path analyses, controlling for the child’s sex and SES, indicated that increased SLD severity predicted an increased number of repeated school grades (*β* = 0.16 and 0.15, *p*s *< *0.01), increased absenteeism (*β* = 0.26 and 0.10, *p*s *< *0.001), and decreased school engagement (*β* = −0.32 and −0.25, *p*s *< *0.001) for multilinguals and English monolinguals, respectively (see Table 6, Model 1). Increased SES corresponded to decreased number of repeated school grades (*β* = −0.08, *p < *0.001), decreased absenteeism (*β* = −0.08, *p < *0.001), and increased school engagement (*β* = 0.11, *p < *0.001) for the English monolinguals; however, these relations were not statistically significant for the multilinguals. The main effect of sex was statistically significant for school engagement (*β* = 0.14, *p < *0.001) and missed school days (*β* = 0.03, *p = *0.01) among the English monolinguals, but not multilinguals^4^. Being a female English monolingual was associated with increased school engagement and increased absenteeism when accounting for the other variables in the model. The group of predictors in the main effects model for academic success accounted for 3–12% (multilinguals) and 2–11% (English monolinguals) of the variance in the outcomes.

**Table 6:** Regression coefficients of academic success by sex, SES, and speech language disorder severity by group.

Model 1	Variable	Repeated any grade				School engagement				Missed school days			
		*β*	*SE*	*p*	*R* ^2^	*β*	*SE*	*p*	*R* ^2^	*β*	*SE*	*p*	*R* ^2^
Multilinguals	Sex	0.01	0.06	0.88	0.03	0.11	0.06	0.06	0.12	0.05	0.01	0.38	0.08
	Socioeconomic status	0.02	0.06	0.75		0.02	0.06	0.68		−0.11	0.01	0.07	
	SLD severity	0.16	0.06	<0.01		−0.32	0.06	<0.001		0.26	0.01	<0.001	
English monolinguals	Sex	−0.02	0.01	0.15	0.03	0.14	0.01	<0.001	0.11	0.03	0.01	0.014	0.02
	Socioeconomic status	−0.08	0.01	<0.001		0.11	0.01	<0.001		−0.06	0.01	<0.001	
	SLD severity	0.15	0.01	<0.001		−0.25	0.01	<0.001		0.10	0.01	<0.001	
Model 2	Variable	*β*	*SE*	*p*	*R* ^2^	*β*	*SE*	*p*	*R* ^2^	*β*	*SE*	*p*	*R* ^2^
Multilinguals	Sex	−0.09	0.17	0.59	0.03	0.27	0.16	0.09	0.12	0.25	0.17	0.13	0.09
	Socioeconomic status	0.02	0.06	0.79		0.03	0.06	0.63		−0.11	0.06	0.09	
	SLD severity	0.05	0.19	0.79		−0.14	0.18	0.43		0.48	0.18	0.008	
	SLD severity × Sex	0.16	0.25	0.53		−0.25	0.23	0.29		−0.30	0.24	0.21	
English monolinguals	Sex	−0.04	0.03	0.18	0.03	0.20	0.03	<0.001	0.11	−0.01	0.03	0.79	0.09
	Socioeconomic status	−0.08	0.01	<0.001		0.11	0.01	<0.001		−0.06	0.01	<0.001	
	SLD severity	0.12	0.04	0.002		−0.17	0.04	<0.001		0.05	0.04	0.22	
	SLD severity × Sex	0.04	0.05	0.40		−0.10	0.05	0.03		0.07	0.05	0.17	

Completely standardized results reported. SLD is speech language disorder.

**Figure 1:**
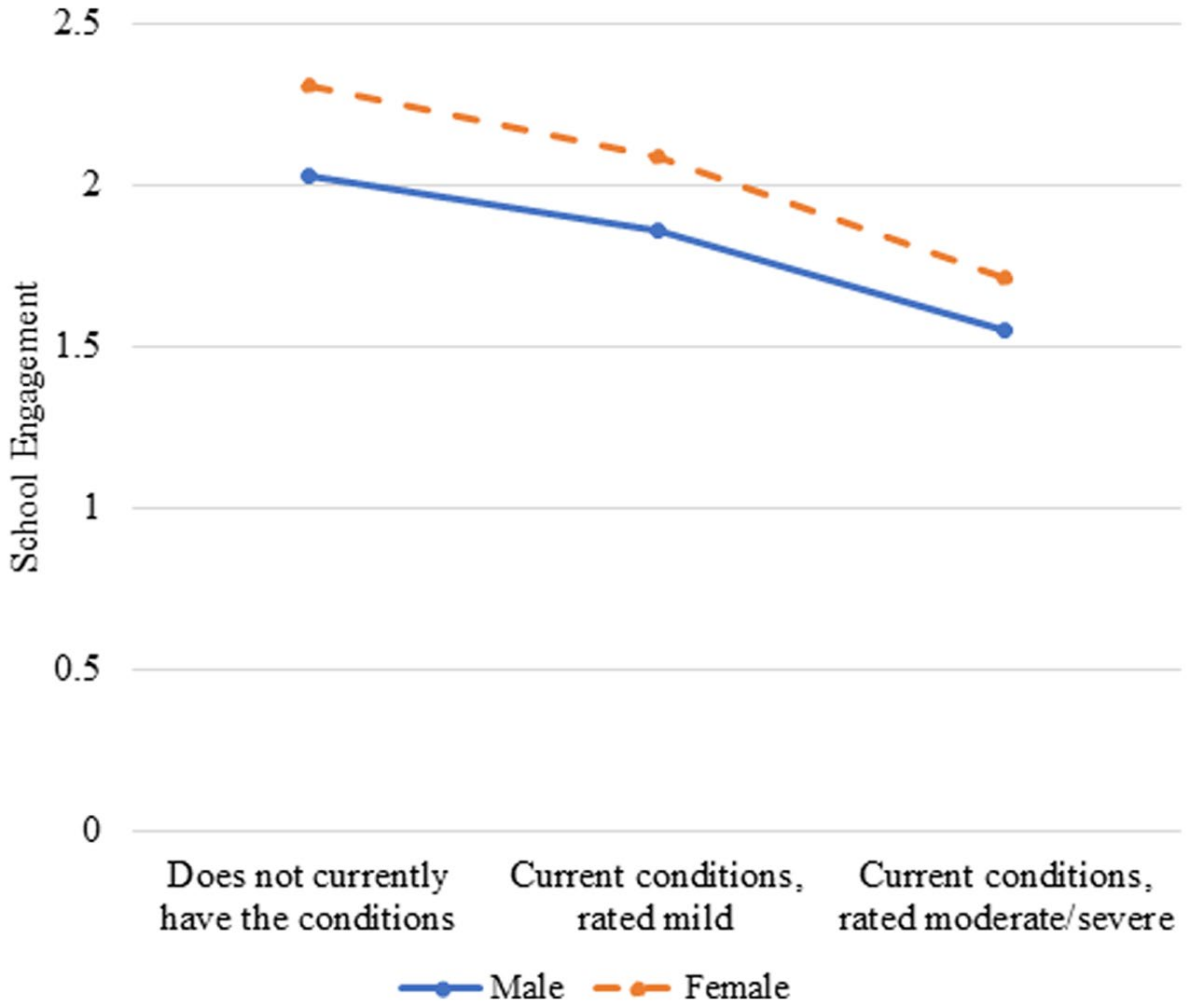
Difficulties making and keeping friends and speech-language disorder severity moderated by sex for English monolinguals.

Model 2, which included the SLD severity by sex interaction, provided evidence of moderation for one outcome among the English monolinguals, school engagement (*β* = −0.10, *p = *0.03; see Table 6). A closer look at the interaction effect suggests that school engagement is higher for females than males across all three categories of SLD severity; however, school engagement decreases more for English monolingual females than males as SLD severity increases (see Figure 1). Notably, the main effect of SES was a statistically significant predictor of the three measures of academic success when accounting for the other variables in the Model for English monolinguals (*β*s = −0.08, 0.11, and −0.06, *p*s < 0.001). The inclusion of the interaction term for each academic outcome, minimally improved the predictive utility of Model 2 over Model 1, accounting for an additional 0–1% and 0–7% of the variance for multilinguals and English monolinguals, respectively.

The results from the tests of measurement invariance for academic success are displayed in Table 5. Both Models (2 and 3), which constrained all parameter estimates to equality across the groups of children, fit the data well. The results suggest that the same general pattern of relations among the regression estimates (*β*), correlations among the three outcomes, intercepts (or mean level of parent reports for each outcome), residual variances and by extension, variances, were evident across the groups of multilinguals and English monolinguals. These findings have implications for how the results are interpreted, which are discussed below.

##### 3.1.5.2. Socioemotional functioning

The results of multigroup path analyses, controlling for the child’s sex and SES, indicated that increased SLD severity predicted increased difficulty making and keeping friends (*β* = 0.34, *p* < 0.001), decreased flourishing (*β* = −0.34, *p* < 0.001), and increased frequency of being bullied (*β* = 0.19, *p* < 0.001) and bullying others (*β* = 0.03, *p* = 0.03) for the English monolinguals (see Table 7, Model 3). Similarly, SLD severity predicted difficulties making and keeping friends (*β* = 0.40, *p* < 0.001) and decreased flourishing (*β* = −0.36, *p* < 0.001) for the multilinguals. Among the English monolinguals, being a female was associated with decreased difficulty making and keeping friends (*β* = −0.08, *p* < 0.001), increased flourishing (*β* = 0.09, *p* < 0.001), and decreased frequency of being bullied (*β* = 0.07, *p* = 0.04). For the multilinguals, neither the child’s sex nor SES was a statistically significant predictor for any of the measures of socioemotional functioning. In contrast, increased SES corresponded increased flourishing (*β* = 0.11, *p* < 0.001) and decreased frequency of being bullied (*β* = −0.08, *p* < 0.001), and bullying others (*β* = −0.04, *p* < 0.001) for the English monolinguals. The group of predictors in the main effects model for socioemotional functioning accounted for 0–18% (multilinguals) and 1–13% (English monolinguals) of the variance in the outcomes.

**Table 7:** Regression coefficients of social–emotional functioning by sex, SES, and speech language disorder severity by group.

Model 3	Variable	Difficulties making and keeping friends				Flourishing				Bullied				Bullied others			
		*β*	*SE*	*p*	*R* ^2^	*β*	*SE*	*p*	*R* ^2^	*β*	*SE*	*p*	*R* ^2^	*β*	*SE*	*p*	*R* ^2^
Multilinguals	Sex	−0.12	0.10	0.25	0.18	0.14	0.12	0.23	0.13	−0.13	0.14	0.35	0.02	−0.01	0.07	0.90	0.00
	Socioeconomic status	0.08	0.06	0.09		0.10	0.05	0.07		−0.10	0.07	0.15		−0.01	0.03	0.86	
	SLD severity	0.40	0.06	<0.001		−0.36	0.06	<0.001		0.11	0.08	0.15		0.01	0.04	0.85	
English monolinguals	Sex	−0.08	0.02	<0.001	0.12	0.09	0.02	<0.001	0.13	0.07	0.03	0.04	0.02	−0.04	0.02	0.08	0.01
	Socioeconomic status	−0.01	0.01	0.64		0.11	0.01	<0.001		−0.08	0.02	<0.001		−0.04	0.01	<0.001	
	SLD severity	0.34	0.01	<0.001		−0.34	0.04	<0.001		0.19	0.02	<0.001		0.03	0.01	0.03	
Model 4	Variable	*β*	*SE*	*p*	*R* ^2^	*β*	*SE*	*p*	*R* ^2^	*β*	*SE*	*p*	*R* ^2^	*β*	*SE*	*p*	*R* ^2^
Multilinguals	Sex	0.02	0.16	0.89	0.18	0.08	0.16	0.63	0.13	−0.24	0.17	0.16	0.03	−0.04	0.17	0.83	0.00
	Socioeconomic status	0.10	0.06	0.08		0.11	0.06	0.07		−0.10	0.06	0.10		−0.01	0.06	0.85	
	SLD severity	0.51	0.17	0.003		−0.32	0.18	0.07		−0.11	0.19	0.56		−0.02	0.19	0.91	
	SLD severity × Sex	−0.14	0.23	0.54		−0.01	0.23	0.97		0.28	0.24	0.26		0.05	0.25	0.85	
English monolinguals	Sex	0.04	0.03	0.20	0.13	0.05	0.03	0.12	0.13	0.06	0.03	0.08	0.03	−0.06	0.03	0.08	0.01
	Socioeconomic status	−0.01	0.01	0.67		0.13	0.01	<0.001		−0.07	0.01	<0.001		−0.05	0.01	<0.001	
	SLD severity	0.46	0.04	<0.001		−0.31	0.04	<0.001		0.17	0.04	<0.001		−0.01	0.04	0.72	
	SLD severity × Sex	−0.15	0.05	0.002		0.01	0.05	0.95		−0.05	0.05	0.33		0.06	0.05	0.24	

Completely standardized results reported. SLD is speech language disorder.

**Figure 2:**
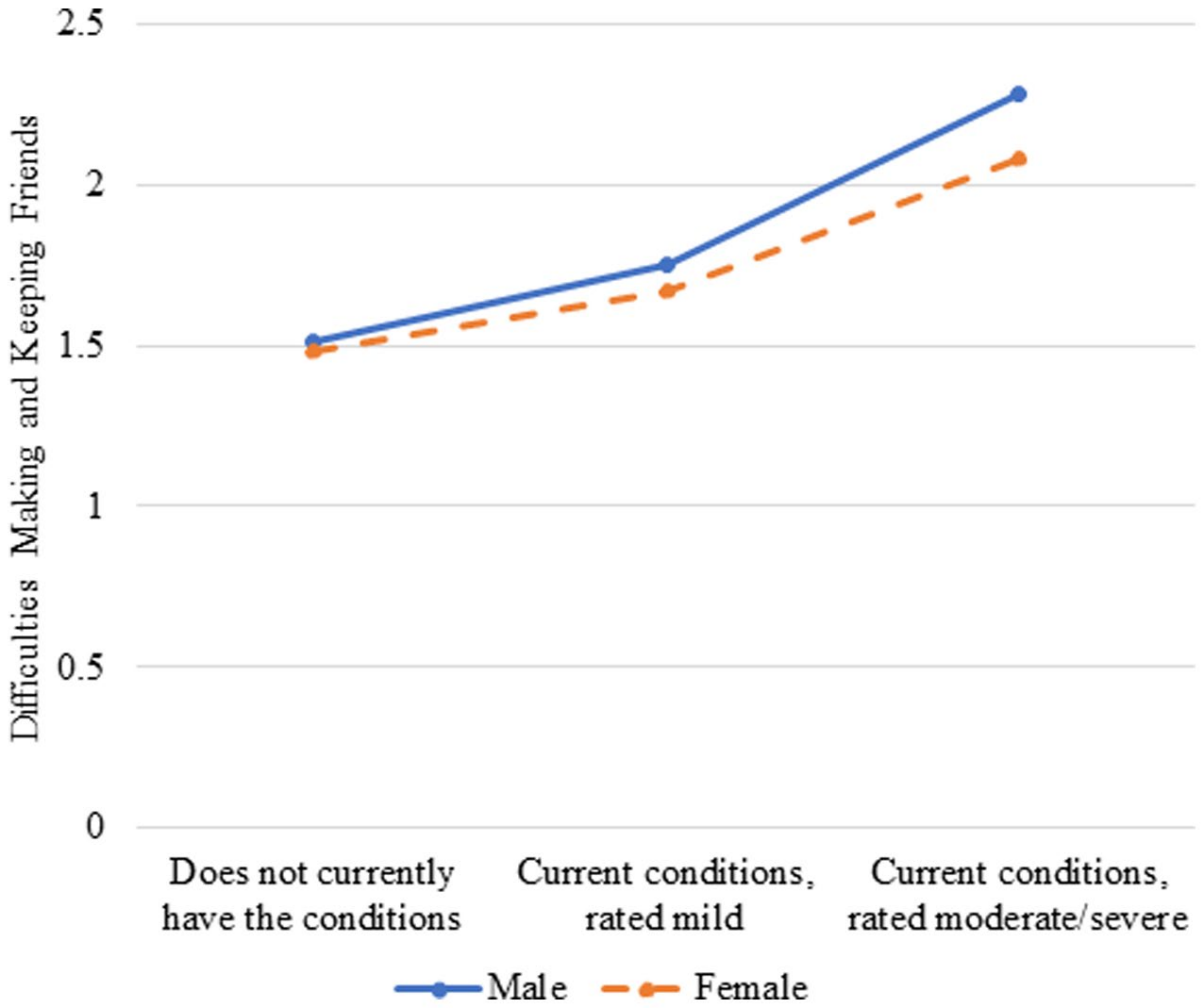
School engagement and speech-language disorder severity moderated by sex for English monolinguals.

Model 4, which included the SLD severity by sex interaction, provided evidence of moderation for difficulty making and keeping friends for English monolinguals (*β* = −0.15, *p* < 0.001) but not multilinguals. A closer look at the interaction indicated that it was more difficult for males to make and keep friends than females regardless of their SLD severity (see Figure 2); however, as SLD severity increased, difficulties making and keeping friends increased more for males than females among the English monolinguals. The inclusion of the interaction term for each socioemotional outcome minimally improved the predictive utility of Model 4 over Model 3, accounting for an additional 0–1% of the variance in the outcomes for both groups of children.

Both tests of invariance indicated that constrained each of the socioemotional functioning Model’s parameter estimates to equality fit the data well (see Table 5, Model 4 and 5). Thus, the general pattern of relations among the predictors and the outcomes as well the mean levels of parent reported difficulties making and keeping friends, flourishing, being bullied, and bullying were equivalent between the groups. These results are discussed below.

## 4. Discussion

To date, few studies have sought to determine the associations between educational success, socioemotional functioning, and SLD severity. In addition, all but one study examining SLDs in children using a large national cohort have focused on monolinguals (Choo et al., 2022). Therefore, the present study used the NSCH publically available dataset to extend this body of research by investigating the impacts of SLD severity on academic success and socioemotional functioning among multilingual and English monolingual children. Since this study is one of the first to include multilingual and English monolingual students, we explored the demographic characteristics (see Table 1), frequency distributions for the observed variables (see Table 2), descriptive statistics (see Table 3), and correlations (see Table 4) to better understand the groups of children before investigating the main analyses (see Tables 5–7). In line with prior reports, the present sample of children with SLDs included larger proportions of males than expected by chance (*cf.* Dodd, 1995; Keating et al., 2001; McLeod et al., 2013; American Speech-Language-Hearing Association [ASHA], 2020; Choo et al., 2022; Irwin et al., 2022). Multilingual children in this study were also more likely to come from lower SES backgrounds (*cf.*, Han, 2012; Callahan, 2013; however, see Padilla and Gonzalez, 2001) and have been identified as showing moderate or severe symptoms (Choo et al., 2022). Multilingual children in this study were more likely to have evidenced some comorbid conditions compared to English monolingual children, although the magnitude of the group differences for comorbid conditions was small. For multilinguals, speaking multiple languages could compound perceptions of language difficulty and make symptoms of some disorders (e.g., Down syndrome, intellectual disability, learning disability, and autism) more prominent, hence, parents may be more likely to report these disorders in their children. Finally, the data indicated that 48 and 63% of multilingual and English monolingual children, respectively, are bullied. This finding is within the range reported (83%) by Davis et al. (2002) and (38%) Hugh-Jones and Smith (1999). In addition, both studies (Hugh-Jones and Smith, 1999; Davis et al., 2002) used child self-report. This suggests that parent report, as used in the present study, yields reliable reports of their children’s socioemotional functioning as well as academic skills. Collectively, the findings from Davis et al. (2002) and Hugh-Jones and Smith (1999) suggest that some children with SLDs, at least those where verbal communication is affected, face high levels of rejection and bullying.

The current study did not find that multilingual children with SLDs are at increased risk of experiencing academic and socioemotional difficulties compared to their English monolingual peers with a SLD. This is in contrast with prior theoretical and empirical findings (Koo et al., 2012; Kang et al., 2014; Zeiders et al., 2016). This difference may be attributed to improvements in the education levels between previous and current immigrants (who are more likely to be highly skilled and educated), which could reduce the difficulties faced by multilingual children in U.S. schools (Krogstad and Radford, 2018). To the extent that progress in educational attainment for second generation immigrants and their children continues, risk for academic and socioemotional difficulties could be further reduced.

While multilingual children evidenced lower levels of school engagement and were less likely to be described as flourishing than monolinguals in the present study, the associations were negligible. Multilinguals were also no more likely to repeat a grade in school or miss more days of school than monolinguals. In fact, multilinguals were described as missing fewer school days, though the association between group and missed school days was negligible. Similarly, multilingual children were no more likely than monolingual children to experience difficulty making and keeping friends. While the associations were negligible, multilinguals were less likely to bully others or have been bullied than monolingual children. In sum, the results of the present study are promising. Prior literature demonstrates that having a SLD makes it more difficult for children to be successful in school (e.g., Bird et al., 1995; Lewis et al., 2000) and to interact with peers and resolve conflicts (e.g., Baker and Cantwell, 1987; Beitchman et al., 1996; Langevin et al., 2009; Dockrell and Howell, 2015). The present study extends these results. Though the parents of multilinguals reported their child’s symptoms as more severe than parents of English monolinguals, multilingualism does not appear to be associated with increased risk for academic or socioemotional difficulties. In the following, we look more closely at SLD severity.

### 4.1. Associations between sex, SES, and SLD severity, academic success, and socioemotional functioning

Being male is a risk factor for SLDs (e.g., for specific language impairment, speech sound disorders, stuttering, voice disorders) in the general population (e.g., Dodd, 1995; Keating et al., 2001; Flax et al., 2003; McKinnon et al., 2007; Harrison and McLeod, 2010; Eadie et al., 2015; American Speech-Language-Hearing Association [ASHA], 2020; Chilosi et al., 2021; Choo et al., 2022). Nonetheless, findings from the present study suggest that although being male is associated with an increased risk for SLDs, sex is not associated with the severity of a child’s SLD symptoms (rated by parents as either “mild” or “moderate/severe”). Further, putative sex-related differences that may decrease the vulnerability to SLDs, did not appear to attenuate SLD severity as the child’s biological sex was not associated with SLD severity in the present study. However, the association between sex and the measures of academic success in our study yielded an interesting set of findings. Namely, being female was associated with increased school engagement and increased absenteeism. One explanation may be that the experience of a SLD and bullying is compounded for girls. Our study found that children with SLDs experience a higher frequency of bullying, and greater difficulty in making and keeping friends. Coincidentally, these two same factors (i.e., bullying and social isolation) are commonly cited by students as reasons for absenteeism, e.g., Malcolm et al., 2003). Further, girls report greater stress and trauma as a consequence of bullying compared to boys (Gruber and Fineran, 2008). In the current study, being female was not associated with greater difficulty in making and keeping friends, higher frequency of being the recipient of bullying, or decreased flourishing. However, girls with SLDs may experience elevated consequences of bullying stress and trauma, increasing their risk of absenteeism. While the association of being female and less difficulty making and keeping friends, less frequently being the recipient of bullying, and increased flourishing were statistically significant for the group of English monolinguals, these associations were just as strong if not stronger for the group of multilinguals. Tests of measurement invariance that constrained the unstructured covariance matrices to equality also suggested that these associations were similar for the groups of children. Past studies report a negative correlation between absenteeism and SES (e.g., Sosu et al., 2021). A novel finding from the present study is the positive correlation between symptom severity and absenteeism, that is, children missed more school days as the severity of their SLD increased. Future studies should investigate whether this relationship is robust.

Finding that SLD severity decreased as SES increased for English monolinguals, but not multilinguals may be due to a combination of socioeconomic, linguistic, and cultural obstacles (Harry, 1992; García Coll et al., 1996; O’Hara, 2003; Blanchett et al., 2009; Peña and Fiestas, 2009; Flores et al., 2010). Namely, parents of multilingual children may be less likely to solicit a professional’s evaluation of their children’s speech and language difficulties, instead preferring to rely on the advice and support of extended families (García Coll et al., 1996; Morgan et al., 2016). In addition, speech-language pathologists (SLPs) in the U.S. are primarily White (92%) and monolingual; only 8.2% of SLPs are multilingual and only 6% of SLPs are Hispanic or Latino. Thus, many SLPs may lack the multilingual training or cultural experience to adequately work with multilingual children (Muñoz et al., 2014; American Speech-Language-Hearing Association [ASHA], 2021, 2022). Further, SLPs may not accurately identify impairments due to the use of assessments designed for children who conform to the dominant culture, not culturally and linguistically diverse populations (Sireci, 2020). Research has shown that children of color are less likely to receive services in early childhood special education (Morgan et al., 2012; Black et al., 2015). Speaking a language other than English as the primary language spoken in the home is associated with decreased access to speech and language services (Morgan et al., 2012, 2016). Having mothers with lower education levels (Wittke and Spaulding, 2018) and lower SES (Bishop and McDonald, 2009) has also been associated with decreased access to speech and language services. Alternatively, the test of measurement invariance suggests that the association between SLD severity and SES was equivalent between the two groups of children. We therefore conducted a posthoc analysis, allowing the correlation between SES and SLD severity to vary between the groups. The nested model (see Table 5, Model 6) fit the data significantly better than the full model (see Table 5, Model 1), *χ*^2^∆(1) = 4.56, *p = *0.03, suggesting that the association between SES and SLD severity differs for multilingual (*r =* −0.16) and English monolinguals (*r =* −0.01). That is, regardless of the mechanism, increased SES appears to be a protective factor against SLD severity for monolinguals but not multilinguals.

### 4.2. Prediction of academic success from SES, sex, and SLD severity

The present findings indicated that academic difficulties increase as the severity of a multilingual and English monolingual child’s SLD intensifies; a finding that is consistent with prior studies among monolinguals only (Bird et al., 1995; Webster et al., 1997). The academic disadvantage that SLDs produce is similar for multilinguals and English monolinguals. As one’s speech and language impairments intensify, the likelihood of repeating a grade in school increases, absenteeism increases, and school engagement decreases. These findings are aligned with results of others who have shown that children with SLDs are less likely to graduate from high school and pursue a college degree than their non-SLD peers, and their college attendance decreases as the severity of their SLD increases (Snowling et al., 2001; Fleming and Fairweather, 2012; Rees and Sabia, 2014). From among the measures of academic success, there was evidence of moderation for school engagement. Namely, females appear to be more engaged in school regardless of the severity of their speech and language impairments; however, school engagement decreases more sharply for females than males as the severity of their SLD intensifies. While this finding was only flagged as statistically significant for the monolingual children, the interaction effect was stronger in magnitude for the group of multilinguals, *β*(*SE*) *=* −0.25(0.23), than monolinguals, *β*(*SE*) *=* −0.10(0.05), though the standard errors suggests that there was more variability in the multilinguals, which is a function of sample size. Given the magnitude of the effects, standard errors, and results from the tests of measurement invariance, it is plausible that these relations are similar for multilingual and English monolinguals.

### 4.3. Prediction of socioemotional functioning from SES, sex, and SLD severity

The results of the present study extend the findings of others (e.g., Davis et al., 2002; Langevin et al., 2009), indicating that difficulties making and keeping friends increases as the severity of the child’s SLD intensifies. The results suggest that children with SLDs appear less interested and curious about learning new things and are less likely to work to finish tasks they start and stay calm and in control when faced with a challenge (i.e., flourishing) as the severity of their SLD intensifies. Such tasks are underpinned by executive functioning. Previous research has found that often children with SLDs have weaker executive functioning relative to children who do not have SLDs, specifically stuttering (Choo et al., 2020). This may be attributed to children with SLDs evidencing comorbid conditions, such as social and generalized anxiety disorder (Iverach et al., 2016), which exacerbates challenges with socializing (Redmond and Rice, 1998) and feelings of embarrassment, frustration, and anger because of their speech and language impairment (Connor et al., 2008). Indeed, the results of the present study provide evidence of comorbid conditions among multilinguals and monolinguals. In addition, our findings extend the results of others in focused on monolinguals (Hugh-Jones and Smith, 1999; Dockrell and Howell, 2015), suggesting that not only is a SLD associated with increased bullying, the frequency of being bullied increases as a child’s speech and language impairments intensify. While being bullied was statistically significant only for the group of monolinguals, it would be wise to consider the magnitude of this relation for multilinguals (*β* = 0.11) and the tests of measurement invariance, suggesting that this relation and inferences drawn from it are equally applicable to multilingual children with SLDs. Lastly, the relation between SLD severity and difficulties making and keeping friends was moderated by sex. Males may find it particularly challenging to make and keep friends as their SLD intensifies.

### 4.4. Implications

Studies of teachers (Daniel and Friedman, 2005) and SLPs (Hammer et al., 2004; Roseberry-McKibbin et al., 2005) generally show that they do not feel prepared to serve multilingual children. Children from nondominant backgrounds experience difficulties in accessing speech and language services (Bishop and McDonald, 2009; Morgan et al., 2012, 2016; Black et al., 2015; Wittke and Spaulding, 2018) as well as historic inequities in the U.S. education system. It is important to rethink how SLPs (and other school personnel) reach out to multilingual families to ensure that information about the potential benefits of speech and language services are made available in a culturally relevant and accessible manner. Parents of multilingual children may benefit from gaining a better understanding of the features of speech and language impairments as well as the consequences of unaddressed impairments (Morgan et al., 2016). Our study has revealed some key insights for parents and those who work with multilingual children. Namely, multilingualism was not associated with increased risk for academic or socioemotional difficulties. Rather, academic and socioemotional difficulties increased as children’s SLD severity intensified. Our findings suggest a bipartite approach to intervention for children with SLD is needed. First, providing children with effective therapy to mitigate SLD symptoms could in turn reduce academic and socioemotional challenges, particularly for children with severe SLD. Second, support for socioemotional difficulties related to bullying and social isolation in the classroom and home could reduce the negative effects of SLD beyond speech and language functioning. At the very least, SLPs, other school personnel, and parents should be aware that males may find it particularly challenging to make and keep friends and females may experience greater absenteeism as the severity of their SLD intensifies.

Our findings that as SLD severity increases, children with SLDs are increasingly likely to repeat a grade, engage in school less, and miss more school should be of assistance to school personnel in charge of school wide applications of behavioral systems and supports. Given the importance of early language development to children’s literacy, math, and science learning (e.g., Duncan et al., 2007; Foster et al., 2022) and the cascading effects of bullying to one’s socioemotional development (Hugh-Jones and Smith, 1999), school personnel must address any problems faced by children with speech and language impairments early. One possible intervention is positive behavioral interventions and supports (PBIS), a school wide application of a three-tiered approach (i.e., universal, selective, indicated) to achieve behavior change (for review, see Weist et al., 2014). PBIS has led to significant reductions in bullying (e.g., Waasdrop et al., 2012) and significant improvements in academic achievement (e.g., Bradshaw et al., 2010). In addition, programming for social emotional learning (SEL) can be implemented within PBIS. SEL emphasizes the perspective that enhancing students’ cognition and emotions are critical for success in school by focusing on developing student’s self-awareness, self-management, social awareness, relationship skills, and responsible decision making (Weist et al., 2014). A meta-analysis by Durlak et al. (2011) indicated that universal SEL led to significantly less emotional distress, fewer negative behaviors, improved school attitudes and behaviors, and better academic performance. Layered into PBIS and SEL, students with speech and language impairments should have access to inclusive education–the process of responding to the diversity of all learners’ needs through increasing participation in learning, cultures, and communities, which covers all children of the appropriate age (UNESCO, 2005)–to ensure that children with SLDs have high quality opportunities to learn and participate in educational programs. Finally, for multilinguals, encouraging bilingual interaction in classrooms and providing instruction that includes linguistically and culturally appropriate topics can support academic success and socioemotional functioning (Krashen, 1982; Cummins, 1997; Ortiz, 2001).

### 4.5. Limitations

While the present study was characterized by several strengths, there were a few limitations. To begin with, the current study employed parent reports for all measures. It is unclear whether SLDs were formally diagnosed, or whether parent recall was accurate. The heterogeneity and variability of speech symptoms may increase the risk of misidentification. Relatedly, experiences associated with the different speech-language disorders are also likely to be highly variable. For example, children with specific language disorder may face different academic and social challenges compared to children who stutter. The present study was not able to disentangle these differences. We suggest this as a future area of investigation. Further, parents’ recall of their child’s SLD may not be accurate and as such, children may be disproportionately (over-or under-) identified in this study. Children were identified as multilingual or monolingual based on parent report of a non-English language spoken within the home. However, the total number of languages children were exposed to, and their degree of proficiency were not determined in the survey. Although bilingual (much less multilingual) education is uncommon in the U.S., it is possible that there are some emergent bilingual children who use English at home and attend language immersion schooling in another language. These children would be considered monolingual in the current study but would likely have some degree of second language exposure (Choo et al., 2022). In addition, SLDs were not operationally defined in the NSCH survey and parents are not trained to identify SLDs. Particularly for multilingual parents, identification may be challenging due to differences in bilingual speech patterns. That is, differences in speech patterns may be identified as a speech-language impairment. Finally, it is important to remember that academic success was based on parent report and may not reflect performance in the classroom. As that may be, other studies have demonstrated that parent reports are rich and reliable sources of information (e.g., Hall and Segarra, 2007; Guiberson et al., 2011; McLeod et al., 2013). Therefore, the use of parent reports does not compromise the internal validity of the study.

A second limitation is that there is no way to determine causality of the relations investigated in the present study. In other words, it cannot be concluded that SLD severity interfered with academic success and socioemotional functioning or if the reverse were true. While we technically have temporal precedence (i.e., parents reported if their children ever had a SLD but reported on academic success and socioemotional functioning during the past 12 months), this assumption is weak. It is possible, for example, that decreased school engagement and missing school led to increased severity of a child’s SLD. Similarly, if a child found it difficult to make and keep friends, they may have fewer opportunities to interact with peers; thereby intensifying the severity of their speech and language symptoms. Most likely the relations identified in the present study are bidirectional, e.g., SLD severity leads to decreased school engagement, increased absenteeism, and increased difficulties making and keeping, which in turn, lead to increased SLD severity. While addressing this concern was not possible with the present dataset, it may be beneficial to use longitudinal data and cross lagged panel models to better understand the relations revealed by the present study.

A final concern is the external validity of this study–the extent to which the results of this study generalize should be interpreted cautiously for a few reasons. One, parents from cultures where disabilities are highly stigmatized may be less likely to identify their child with a disorder while parents who place a higher value on verbal ability may be more likely to report SLD as a concern (Choo et al., 2022). Further, SLDs were not operationally defined in the NSCH survey, consequently, it is plausible that children with other developmental disorders (e.g., attention-deficit/hyperactivity disorder) were misidentified with a SLD (Choo et al., 2022). Lastly, some parents may be apprehensive about sharing health information and chose not to complete the NSCH survey. As a result, it could be beneficial to replicate this study with another dataset to help determine the generalizability of the findings. As that may be, the present results are consistent with the work of others and limitations do not take away from the findings.

### 4.6. Conclusion

The present study is among the first to focus on the intersection of SLDs and multilingualism. The findings suggest more similarities than differences between multilingual and monolingual children who have speech and language impairments. Notably, SLD severity decreased as monolinguals’ SES increased, however this was not the case for multilingual children. Being female was associated with increased school engagement even though females missed more days of school than males for the monolinguals, though the direction and magnitude of these regression effects were similar for multilinguals. Being female was also associated with fewer difficulties making and keeping friends and increased flourishing for the monolinguals. Again, the direction and magnitude of these regression effects were similar for multilinguals. Regarding bullying, being female and monolingual was associated with increased frequency of being bullied but being female and multilingual was not. Thus, there was evidence in support of the putative effects of being female, though the findings were mixed. The results of this study highlight the need for school personnel to consider a child’s SLD severity. Specifically, increased severity in one’s speech and language impairments correspond to increased frequency of repeating a grade, absenteeism, difficulty making and keeping friends, and decreased school engagement and flourishing for both groups of children. Increased severity of one’s speech and language impairment was also associated with increased frequency of being teased and bullied for monolinguals, though we attribute the lack of statistical significance for multilinguals to their smaller sample size. The results of the present study could inform treatment and the development of intervention programs for SLDs.

## Data availability statement

Publicly available datasets were analyzed in this study. This data can be found here: https://www.childhealthdata.org/browse/survey?s=2&y=43&r=1.

## Ethics statement

For the current study ethical review and approval, and written informed consent, were not required in accordance with the local legislation and institutional requirements. The NSCH data on which this study is based were reviewed by the NCHS Research Ethics Review Board (ERB) and the Abt Associates Institutional Review Board (IRB). The patients/participants provided their written informed consent to participate in this study.

## Author contributions

AC and SS developed the theoretical framework. AC prepared the data. MF and SS planned the data analyses. MF completed the data analyses. All authors discussed the results and wrote the manuscript, which was a collaborative effort and conceived of the presented study and drafted the manuscript, which was led by MF.

## Conflict of interest

The authors declare that the research was conducted in the absence of any commercial or financial relationships that could be construed as a potential conflict of interest.

## Publisher’s note

All claims expressed in this article are solely those of the authors and do not necessarily represent those of their affiliated organizations, or those of the publisher, the editors and the reviewers. Any product that may be evaluated in this article, or claim that may be made by its manufacturer, is not guaranteed or endorsed by the publisher.

## Footnotes

^1^In the U.S., English language learners are students who meet Title III criteria of the Every Student Succeeds Act of 2015 to receive supplemental services in the public school system to improve their English language proficiency and academic achievement.

^2^IRB approval was not required for the present study because it only involves the study of existing, publicly available data that was prepared with the intent of making the data available for the public. The participants cannot be identified, directly or through identifiers linked to the participants.

^3^Not displayed in Table 1.

^4^It should be noted that the exact *p* value for the child’s sex predicting school engagement was 0.057 for the multilinguals, which is often reported as marginally significant.
